# Fibrotic response after Baerveldt glaucoma drainage device surgery: an initial sight-threatening sign in eyes with refractory childhood glaucoma

**DOI:** 10.1007/s10384-026-01357-w

**Published:** 2026-05-02

**Authors:** Rumi Kawashima, Kenji Matsushita, Masako Kurashige, Takahiro Matsui, Yuichi Motoyama, Takahiro Fujino, Eiichi Morii, Kohji Nishida

**Affiliations:** 1 Department of Ophthalmology, Graduate School of Medicine, The University of Osaka, Yamadaoka 2-2, Suita, Osaka 565-0871 Japan; 2 Department of Pathology, Graduate School of Medicine, The University of Osaka, Osaka, Japan; 3 Integrated Frontier Research for Medical Science Division, Institute for Open and Transdisciplinary Research Initiatives, The University of Osaka, Osaka, Japan

**Keywords:** Childhood glaucoma, Baerveldt glaucoma implant, Refractory glaucoma, Fibrosis

## Abstract

**Purpose:**

We report the pathological analysis of a postoperative fibrotic reaction as an initial sign of a sight-threatening complication observed during the 2-year outcomes follow up of Baerveldt glaucoma implant (BGI) surgery in eyes with refractory childhood glaucoma.

**Study design:**

Retrospective case series.

**Methods:**

Fifteen eyes of 10 patients with refractory childhood glaucoma underwent BGI surgery between 2013 and 2020 at the University of Osaka Hospital. Patients were followed for a minimum of 2 years postoperatively. We investigated the postoperative complications, and histopathologically analyzed the specimens obtained from three eyes with postoperative fibrous membranes on the irises.

**Results:**

Nine eyes (60%) had a postoperative fibrous membrane around the tube or on the iris close to the tube, whereas two eyes developed a tractional retinal detachment. The pathological results indicated the tissues were eosinophilic fibrous scar tissues that did not have inflammatory or neoplastic lesions.

**Conclusion:**

BGI surgery was effective in patients with refractory childhood glaucoma. However, clinicians should be alert to the potential for development of fibrotic ocular inflammation in refractory childhood glaucoma eyes.

**Supplementary Information:**

The online version contains supplementary material available at 10.1007/s10384-026-01357-w.

## Introduction

Childhood glaucoma must be diagnosed and treated as early as possible to prevent childhood blindness and protect patients’ future vision. Medical therapy is usually difficult because of poor efficacy, difficulties with regular administration, and serious systemic adverse effects [1]. The main treatment for primary or secondary childhood glaucoma is surgery because the anatomies of trabecular meshwork and Schlemm’s canal are abnormal [2]. The first surgical choice is angle surgery (goniotomy or trabeculotomy), but, in primary childhood glaucoma approximately 20% of surgery will eventually fail [3]. In addition, the success rate of angle surgery for more advanced or severe cases of childhood glaucoma is poorer [4]. For those refractory cases, other surgical options include filtration surgery, cyclodestructive procedures, or glaucoma drainage device (GDD) implantation. The stronger scarring response and a higher rate of endophthalmitis in children result in lower success rates of filtration surgery compared with adults [5, 6]. The effect of the cyclodestructive procedure in children is also limited and sometimes causes phthisis and vision loss [7]. Although GDD implantation is effective in refractory childhood glaucoma [8] and has a higher success rate than trabeculectomy with mitomycin C [9], GDD-related complications or several severe sight-threatening complications may occur over the course of a long lifetime [10]. The factors that are significant in surgical failure remain unclear. The postoperative fibrotic reaction, reported previously, might cause tube malposition [2, 8, 13] but the clinical relevance is unknown in the development of severe complications. This report is the first to describe the pathological analysis of a postoperative fibrotic reaction and the relationship with a sight-threatening complication.

## Materials and methods

This was a retrospective study of patients with refractory childhood glaucoma. We treated 15 eyes (10 cases) with Baerveldt glaucoma implant (BGI) surgery performed by one surgeon at the Department of Ophthalmology, the University of Osaka Hospital. We analyzed patients with childhood glaucoma who were younger than 20 years with a minimal follow-up period of 2 years after BGI surgery and a reliable ophthalmic examination. The guidelines of the World Glaucoma Association Consensus were the basis for glaucoma diagnoses [11]. The institutional review board of The University of Osaka Hospital approved this study (approval number 17330-4), which adhered to the tenets of the Declaration of Helsinki.

### Surgical technique

One glaucoma specialist (KM) performed all surgery with all patients under general anesthesia as reported previously [12]. He performed a conjunctival peritomy and dissected the conjunctiva and Tenon’s capsule about 90 degrees at the site of the planned implantation of the model BG 101-250 in 13 eyes or the model BG 101-350 in two eyes (Abbott Medical Optics). He implanted the shunts in the inferotemporal quadrant in eight patients and in the superotemporal quadrant in seven patients. The endplate was sutured to the sclera 10 mm posterior to the limbus. The tube was inserted into the anterior chamber in eight phakic eyes, into the sulcus using a 23-gauge needle in six aphakic eyes, and into the posterior chamber in one aphakic eye. The tube was completely ligated at a distance of 2 mm before the plate using an 8-0 Vicryl suture (Ethicon, Cincinnati) and two Sherwood slits were made into the tube. The tube was covered with a donor scleral patch. Finally, the conjunctiva was sutured closed. Intraoperatively, neither triamcinolone nor mitomycin C was administered. Postoperatively, topical levofloxacin and betamethasone were started at a dosage of four times daily and gradually tapered, and treatment was continued for 3 months to 2 years. In cases of hypotony, the frequency of topical betamethasone was increased to six times daily, and topical atropine was additionally prescribed.

### Data collection

We collected data that included age, gender, ocular and systemic diagnoses, history of ocular surgery, and surgical details and complications. On the day of surgery, we performed examinations with the patients under anesthesia that included measurements of the corneal diameter, intraocular pressure (IOP), and axial length using A-mode ocular biometry, and intraocular examinations using B-mode ophthalmic ultrasonography, ultrasound biomicroscopy, and RetCam 3 Ophthalmic Imaging System (Clarity Medical Systems). Postoperatively, the patients were examined on weeks 1, 2, 4, and 6, and months 2, 3, 6, 12, and 24 after BGI surgery. The IOP was measured if possible, at each visit using Goldmann applanation tonometry. Most IOP measurements were obtained using iCare (Vantaa) with the patients under sedation or Tonopen (Reichert, Depew) tonometers under general anesthesia. The visual acuity was determined using appropriate measures regarding age or development. At each visit, the patients underwent slit-lamp microscopy and fundus examination, and B-mode ophthalmic ultrasonography if needed.

### Pathological analysis

In three eyes that had anterior chamber inflammation after BGI surgery, we removed the fibrous membrane from around the tube or on the iris during other surgery and analyzed them using hematoxylin and eosin (H&E) staining.

## Results

### Patient characteristics and postoperative complications

Table 1 shows the patient characteristics. The study included 15 eyes of 10 patients, of whom 9 (90.0%) were boys. The mean age at surgery was 6.8 ± 6.1 years. Secondary childhood glaucoma accounted for most cases (13 eyes, 86.7%), with retinopathy of prematurity being the most common underlying condition. All the patients had undergone multiple prior ocular surgery, with a mean of 2.9 ± 1.1 procedures per eye. Conventional trabeculotomy was the most frequent previous surgery, followed by pars plana vitrectomy. The Baerveldt tube was inserted into the anterior chamber in most eyes, while sulcus and pars plana insertions were performed in selected cases. The mean preoperative intraocular pressure was 31.7 ± 9.0 mmHg, and the mean preoperative glaucoma medication score was 5.7 ± 1.7. During the observation period, the postoperative complications included vitreous hemorrhage (n=4, 26.7%), hyphema (n=4, 26.7%), choroidal detachment (n=3, 20.0%), partial tube obstruction due to fibrous membrane (n=1, 6.7%), need for additional IOP-lowering surgery (n=3, 20.0%), anterior chamber fibrotic reaction (n=9, 60.0%), and tractional retinal detachment (n=2, 13.3%) (Table 2). All patients with vitreous hemorrhages and hyphema recovered spontaneously. Only one with choroidal detachment required injection of an ophthalmic viscosurgical device and experienced prolonged hypotony lasting approximately 2 weeks, which subsequently resolved. The tube was partially obstructed by fibrin membrane in one patient 1 month after BGI surgery with sulcus tube insertion. Three eyes with anterior tube insertion required an additional IOP-lowering surgery, i.e., one more BGI surgery. Table 3 shows the characteristics of patients with postoperative fibrotic reaction. The fibrous membrane occurred in the anterior chamber from 1 week to 9 months after BGI surgery in five eyes with anterior tube insertion, three eyes with sulcus insertion, and one eye with posterior insertion. One aphakic patient with Marfan syndrome who had undergone surgery for vitreous hemorrhage developed prolonged postoperative hypotony and subsequently experienced inoperable tractional retinal detachment at 6 months postoperatively (Case 7). Additionally, in the left eye of Case 2, proliferative vitreoretinopathy (PVR) was identified intraoperatively during cataract surgery performed approximately two years after the Baerveldt implantation.

**Table 1:** Patient demographic data

Parameter	No. (%)
Sex	
Men	9 (90.0)
Women	1 (10.0)
Age at surgery (years)	6.8 ± 6.1
Type, number eyes	
Primary open-angle childhood glaucoma	2 (13.3)
Secondary glaucoma	13 (86.7)
Retinopathy of prematurity	5 (33.3)
Marfan syndrome	2 (13.3)
Stickler syndrome	2 (13.3)
Sturge-Weber syndrome	1 (6.7)
Craniosynostosis	1 (6.7)
Post-cataract surgery	1 (6.7)
Post-trauma	1 (6.7)
No. of previous ocular surgeries	2.9±1.1
1	2 (13.3)
2	3 (20.0)
3	5 (33.3)
4	5 (33.3)
Previous surgery	
Conventional trabeculotomy	12 (80.0)
Trabeculectomy	4 (26.6)
Pars plana vitrectomy	6 (39.9)
Pars plana lensectomy	3 (20.0)
Phacoemulsification	1 (6.7)
Phacoemulsification and intraocular lens implantation	1 (6.7)
Iridectomy	1 (6.7)
Scleral buckle encircling	2 (13.3)
Placement of a corneoscleral suture for trauma	1 (6.7)
Tubal insertion site	
Anterior chamber	10 (66.7)
Sulcus	4 (26.7)
Pars plana	1 (6.7)
IOP preoperatively (mmHg)	31.7 ± 9.0
Preoperative glaucoma medications score (points)	5.7 ± 1.7

**Table 2:** Postoperative complications of BGI surgery

Complication	No. (%)
	14 (93.3)
Hyphema	4 (26.7)
Vitreous hemorrhage	4 (26.7)
Choroidal detachment	3 (20.0)
Partial tube obstruction	1 (6.7)
Additional IOP-lowering surgery	3 (20.0)
Anterior chamber fibrotic reaction	9 (60.0)
Early (≦ 1 month)	3 (20.0)
Late (> 1 month)	6 (40.0)
Tractional retinal detachment	2 (13.3)

**Table 3:** Characteristics of subjects with postoperative intraocular fibrotic reaction

Case	Type of glaucoma	Age (years)	Previous glaucoma surgeries	Weeks to intraocular fibrotic reaction	Site of tube insertion	Postoperative inflammatory findings
1	ROP	1.4	PPVx2, LSV, TLOT	6	Anterior	Pupil deviation
2	Primary	3.6	TLOTx3	24	Anterior	Iris atrophy
	Primary	3.6	TLOTx3	16	Anterior	Synechia, cataract, iris atrophy, Proliferative vitreoretinopathy
3	Sturge-Weber	19.4	TLOTx3, TSCPC	8	Anterior	Transient hypotony, Synechia
4	ROP	10.7	PPL, PPV, encircling	1	Sulcus	VO, epiretinal fibrotic membrane
	ROP	10.7	PPL, PPV, encircling	1	Sulcus	VO, epiretinal fibrotic membrane
5	Crouzon	12.1	PEA+IOL+AvitTSCPCx2, TLOTx2	4	Sulcus	Transient hypotony, Partial tube obstruction
6	ROP	3.2	Iridectomy, PEA	8	Posterior	Vitreous cavity fibrotic membrane
7	Marfan	3.0	TLOT, TLEC	36	Anterior	Transient hypotony, cataract
	Marfan	3.0	TLOT, TLEC, PPV, PPL	24	Anterior	Prolonged hypotony for 2 weeks, Proliferative vitreoretinopathy

*ROP* retinopathy of prematurity, *PPV* pars plana vitrectomy, *LSV* lens-sparing vitrectomy, *TLOT* conventional trabeculotomy, *TSCPC* transscleral cyclophotocoagulation, *TLEC* trabeculectomy, *RD* retinal detachment, *PEA* phacoemulsification, *PPL* pars plana lensectomy; *VO* vitreous opacity, *IOL* intraocular lens, *Avit* anterior vitrectomy

### Pathological analysis of postoperative fibrous membranes

Nine eyes (60%) had postoperative anterior chamber inflammation with a fibrous membrane that developed from 1 to 36 weeks after BGI surgery. We treated these eyes with steroid eyedrops, but the inflammation persisted. We removed the fibrous membrane around the tube or on the iris in three eyes during other surgery and analyzed them using H&E staining.

**Fig. 1:**
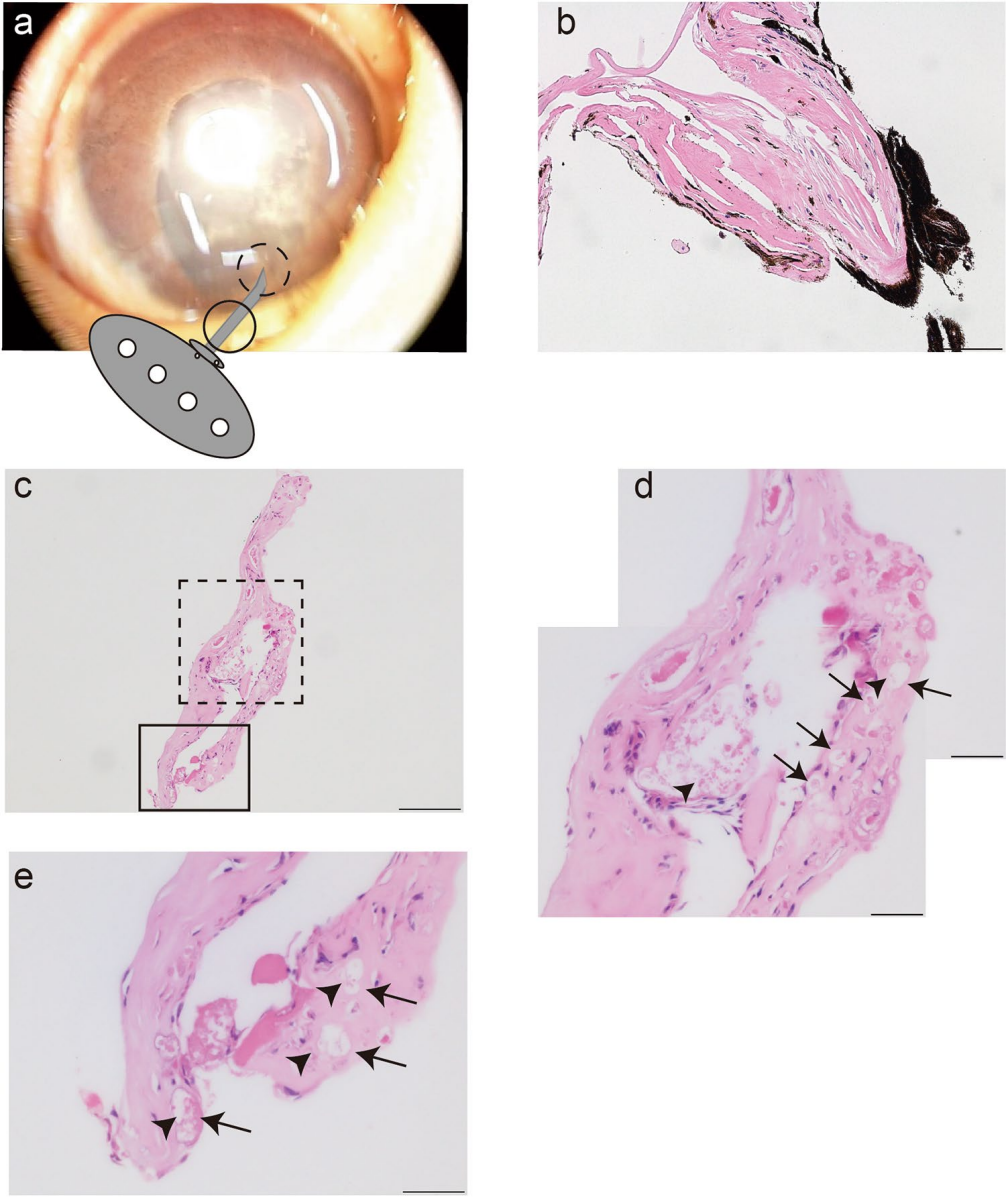
The right eye of a 1-year-old girl who underwent Baerveldt glaucoma implant (BGI) surgery for secondary childhood glaucoma because of retinopathy of prematurity. **a** The BGI plate is in the inferotemporal quadrant and the tube is inserted into the anterior chamber. A severe fibrous membrane developed after BGI surgery on the iris around the tube (dotted circle) and around the tube near the BGI plate (circle). **b**–**e** H&E staining of the removed fibrous membrane: **b** in the dotted circle; **c** in the circle; **d** high-magnification image in the dotted square; and **e** high-magnification image in the square. The arrows indicate the vacuoles and the arrowheads indicate the eosinophil granules. Scale bars, 50 µm in (**b**), (**d**), and (**e**); and 200 µm in (**c**)

Case 1 was of the right eye of a 1-year-girl with secondary glaucoma resulting from retinopathy of prematurity. The fibrous membrane emerged around the tube inserted into the anterior chamber and on the iris 6 weeks postoperatively. The iris became atrophic, the pupil was positioned abnormally in relation to the tube, and the cataract progressed. We removed the fibrous membrane on the iris, around the tip of the tube, and around the tube near the BGI plate during cataract surgery (Fig. 1a).

**Fig. 2:**
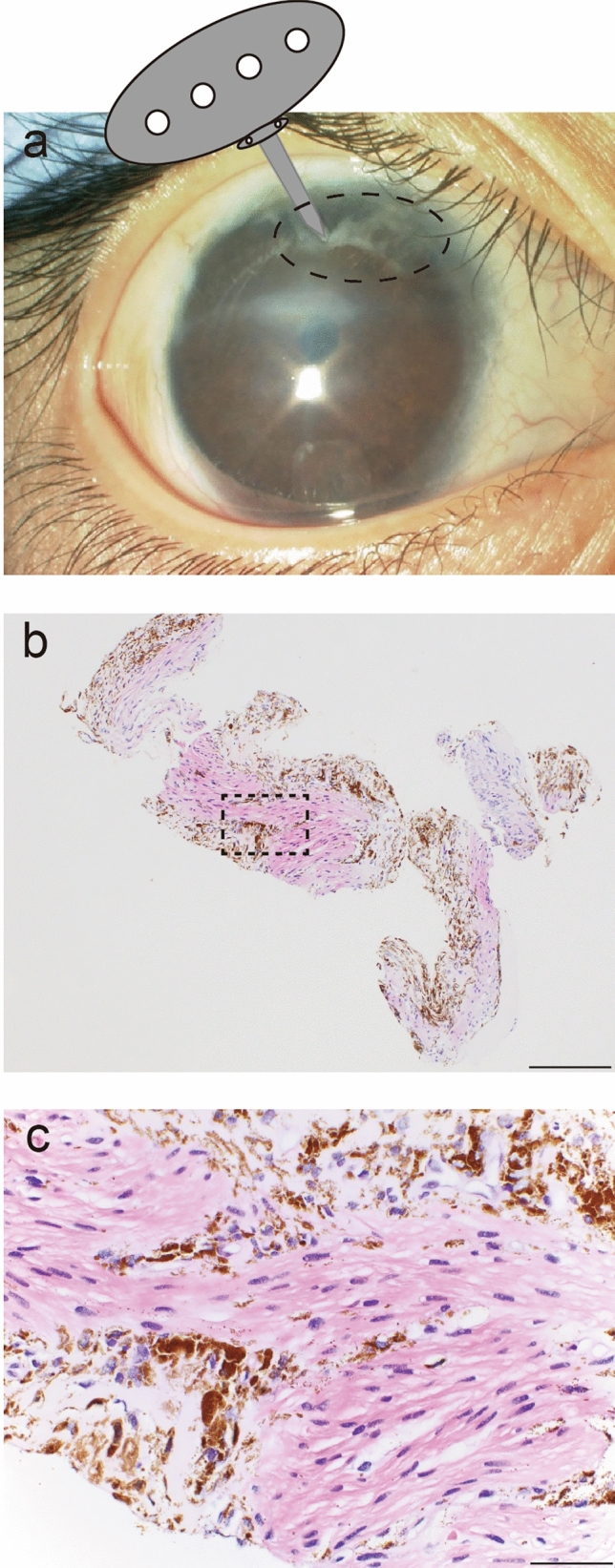
The right eye of a 3-year-old boy who underwent BGI surgery for primary childhood glaucoma. **a** The BGI plate is in the superotemporal quadrant and the tube is inserted into the anterior chamber. The dotted circle indicates the tissue around the tip of the tube. **b**, **c** H&E staining of the excised fibrous membrane: **b** in the dotted circle in (**a**); **c** high-magnification image in the dotted square in (**b**). Fibroblasts proliferated diffusely or in bundles, with little inflammatory cell infiltration. Scale bars, 200 µm in (**b**) and 50 µm in (**c**)

**Fig. 3:**
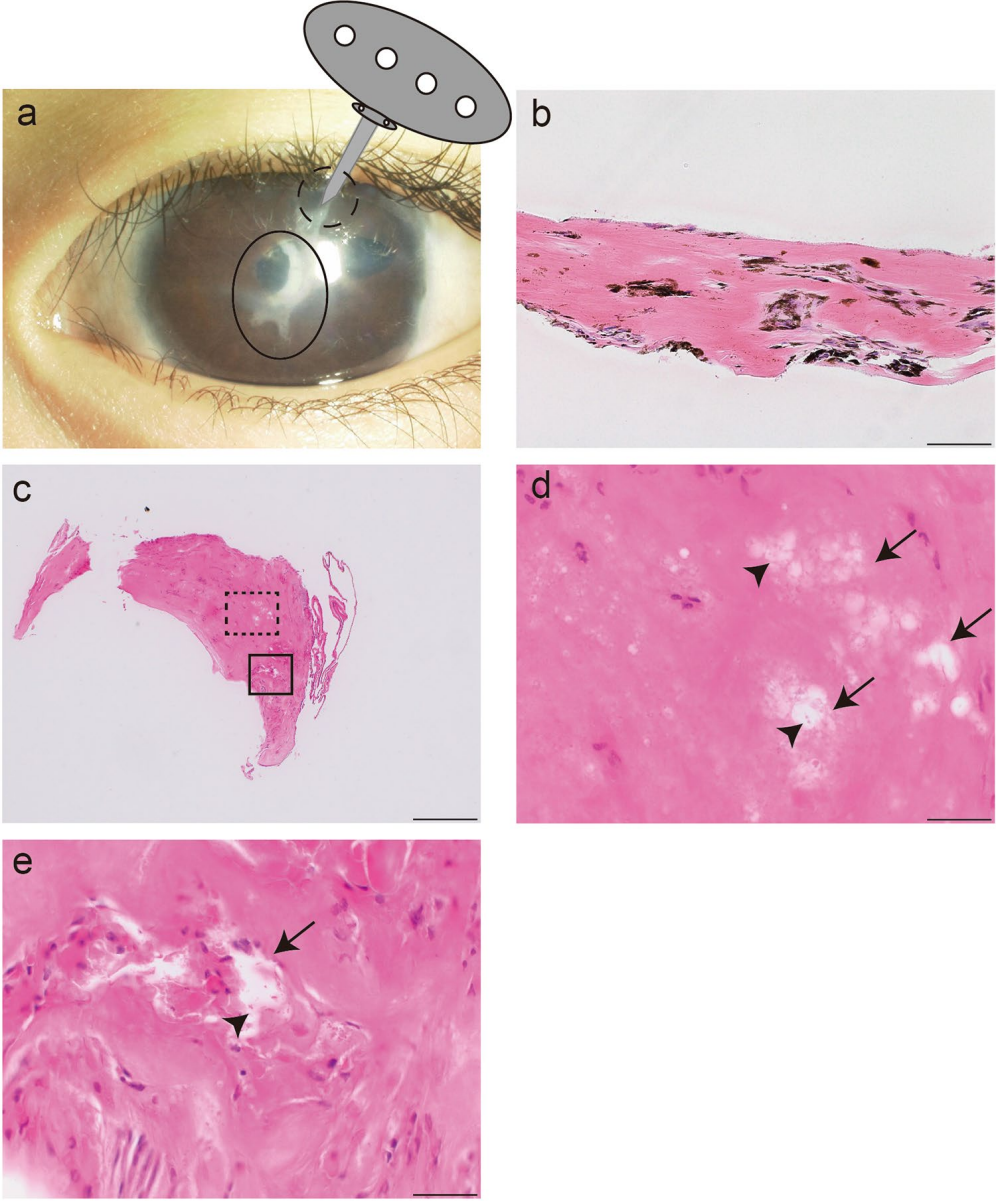
The left eye of a 3-year-old boy who underwent BGI surgery for primary childhood glaucoma. **a** The BGI plate is in the superotemporal quadrant and the tube is inserted into the anterior chamber. The dotted circle indicates the tissue around the tip of the tube and the circle indicates the fibrous membrane on the iris in the anterior chamber. **b**–**e** H&E staining of the excised fibrous membrane: **b** the dotted circle in (**a**); **c** the circle in (**a**); **d** high-magnification image in the dotted square in (**c**); and **e** high-magnification image in the square in (**c**). Hyalinized collagen fibers were diffusely deposited, and vacuoles containing acidophilic granules were observed sporadically. The arrows indicate the vacuoles and the arrowheads indicate the eosinophil granules. Scale bars, 100 µm in (**b**), 500 µm in (**c**), and 50 µm in (**d**) and (**e**)

Case 2 was of both eyes of a 3-year-old boy with primary childhood glaucoma treated with three previous trabeculotomies. The right eye had iris atrophy and a fibrous membrane on the iris and anterior capsule 6 months postoperatively (Fig. 2a); the left eye had posterior synechia, cataract, iris atrophy, and a fibrous membrane around the tip of the tube 4 months postoperatively (Fig. 3a). During cataract surgery, fibrous membranes were removed and analyzed in both eyes. In the left eye, PVR was identified intraoperatively, necessitating additional pars plana vitrectomy. The pathological results showed that all tissues were eosinophilic fibrous scar tissues, including eosinophil granules and vacuoles, that did not have inflammatory or neoplastic lesions (Figs. 1b–e, 2b and c and 3b–e). These findings indicate that mature scar tissue rich in collagen and other proteins was secreted by fibroblasts.

## Discussion

We generally perform surgery as the initial management of childhood glaucoma. Angle surgery, the gold standard, is often performed and is associated with a low complication rate. However, many varieties of secondary childhood glaucoma, such as Sturge-Weber syndrome, aphakia, aniridia, and retinopathy of prematurity, have lower success rates of angle surgery and trabeculectomy because of strong scarring or sight-threatening postoperative complications [2]. For this reason, GDD implantation is a good alternative. The mean IOP and IOP-lowering medication score after BGI surgery significantly decreased, respectively, from 31.7 ± 9.0 to 19.3 ± 8.2 mmHg and from 5.7 ± 1.7 to 1.2 ± 1.8 at 2 years (Online Resource 1). The success rates were 80.0% at 12 months and 60.0% at 24 months based on definition 1, and 60.0% at 12 months and 53.3% at 24 months based on definition 2 (IOP ≧22 mmHg [definition 1] or ≧17 mmHg [definition 2] with or without medication use at two consecutive follow-up visits) (Online Resource 2). These success rates are lower than in previous studies possibly because the current cases had a higher rate of secondary childhood glaucoma than in previous reports (86.7% vs. 47.9% [13], 36.4% [14], or 31.1% [15]). Another reason was that the number of previous ophthalmic surgical procedures in this study was higher than in previous reports (2.9 vs. 1.6 [13] or 1.0 [14]). In another study that reported the outcomes of BGI surgery for refractory childhood glaucoma, the secondary glaucoma rate, 62.9%, and the previous number of surgical procedures, 1.7, showed that the success rates (IOP <22 mmHg, ≧5 mmHg with or without medication use) were similar (80% at 12 months and 67% at 24 months) [8]. Another study reports that the success rates (IOP <22 mmHg, ≧5 mmHg with or without medication use) of a pars plana BGI surgery for refractory childhood glaucoma were 85% at 12 months and 81% at 24 months, i.e., the secondary glaucoma rate was 96.7%, the number of previous surgical procedures was 2.1, and the aphakic eye rate was 80% [16]. Taken together, our study shows that BGI surgery for refractory childhood glaucoma is effective.

In the current study, the rate of postoperative complications related to the aqueous shunt plate or the tube specific to GDD was similar to those reported previously [8, 15–17]. The rates of hyphema, vitreous hemorrhage, and choroidal detachment because of hypotony were higher than reported previously [8, 15–17], but all incidents were temporary. The severe complication in the current study was tractional retinal detachment in two eyes (13.3%). Refractory glaucoma or pars plana tube insertion can cause vision-threatening complications [8, 16, 17]. We also show that the development rate of fibrous membrane on the iris or around the tube was high after BGI surgery, without apparent postoperative strong anterior segment inflammation. Those fibrotic tissues were rich in proteins such as collagen secreted by fibroblasts and were neither inflammatory nor neoplastic in nature. The higher incidence of anterior chamber fibrotic complications observed in this study may reflect differences in patient characteristics, including a high prevalence of secondary glaucoma and multiple prior surgeries, as well as differences in how fibrotic reactions were recognized and reported in previous studies. Children have a robust healing response, which may lead to a higher rate of fibrosis and scarring [5]. This response can lead to the failure of filtering surgery in childhood glaucoma. The precise cause or mechanism of the strong healing response is unknown. In particular, the strong intraocular inflammation after GDD implant surgery in children has not been investigated until now. It is well known that fibrotic encapsulation around the plate causes GDD implant failure. Furthermore, fibrovascular ingrowth into the valve chamber of the AGV causes late AGV failure in children [18] and adults [19]. Trigler et al. hypothesize that the enhanced healing reaction in children compared with adults may explain the high prevalence of AGV fibrovascular ingrowth [18]. In pediatric glaucoma eyes after BGI surgery, the frequencies of encapsulation, uveitis, tube occlusion or malposition, and retinal detachment are higher compared with adults [9, 13–15, 20]. Six of our nine cases with anterior chamber fibrotic reaction occurred after postoperative week 6, coinciding with tube suture ligation release. These results may indicate that BGI, a non-valved device, has backward flow of fluid from the BGI plate into the eye, not just outflow from inside the eyeball to the tube, and inflammatory mediators in the intracapsular fluid around the BGI plate, which stimulate the fibroproliferative process flow into the eye. In particular, a severe fibrotic reaction may cause a sight-threatening complication, such as a tractional retinal detachment. We speculate that fibrotic proliferation extended from the anterior chamber into the vitreous cavity, generating traction that led to ciliary body detachment and the formation of retinal tear at the ora serrata, ultimately resulting in the development of PVR. However, the cellular and molecular differences between fibrotic tissue formed around the Baerveldt plate and intraocular fibrotic tissue remain unclear, and further histopathological or biochemical studies are required to clarify this mechanism.

In addition, postoperative hypotony may represent another potential contributor to postoperative inflammation and fibrotic responses. In the present study, postoperative intraocular pressure of ≤5 mmHg was observed in three of the nine cases with anterior chamber fibrotic reaction, although hypotony in these cases resolved within one week and was not prolonged. However, one patient experienced prolonged postoperative hypotony persisting for more than two weeks and later developed tractional retinal detachment. Although transient hypotony resolved within a short period in most cases, prolonged hypotony may further exacerbate postoperative inflammation, potentially promoting fibrotic reactions. Therefore, early recognition and management of hypotony are crucial to minimize postoperative inflammatory and fibrotic complications. In our practice, early intensification of anti-inflammatory therapy and the addition of atropine eye drops were implemented promptly when hypotony was detected. In addition, selecting a valved glaucoma drainage device, such as the Ahmed glaucoma valve, may represent one possible strategy to reduce the risk of postoperative hypotony in selected cases.

On the other hand, Miyako et al. report that fibrous proliferative membrane in the anterior chamber after Ahmed glaucoma valve implantation occurred following micropulse ciliary photocoagulation in a pediatric glaucoma patient. And they speculate that this reaction might be caused by the silicone tube of the Ahmed glaucoma valve [21]. We do not yet know whether prolonged hypotony, a foreign body reaction to materials, or specific wound healing in children causes the development of strong fibrous membranes, but this condition is similar to aniridia fibrosis syndrome [22], and we propose Pediatric Hyperreactive Anterior Segment Fibrosis (PHASF) as a novel designation to classify this condition. Thus, we show the need for follow-up and careful management of postoperative inflammation in the anterior and posterior ocular segments in patients with refractory childhood glaucoma after BGI surgery.

The current study has some limitations. First, this study was retrospective and the sample size was small. Second, the surgical technique varied depending on the case. Third, the findings of this study may not be generalizable to other glaucoma implant devices with different designs or materials.

In conclusion, BGI surgery was effective in patients with refractory childhood glaucoma. However, clinicians should be alert to the potential for development of fibrotic ocular inflammation in refractory childhood glaucoma eyes.

## Supplementary Information

Below is the link to the electronic supplementary material.

Supplementary file1 (PDF 375 kb)

## References

[1] Yu Chan JY, Choy BN, Ng AL, Shun JW. Review on the management of primary congenital glaucoma. J Curr Glaucoma Pract. 2015;9:92–9. 26997844 10.5005/jp-journals-10008-1192 PMC4779948

[2] Chen TC, Chen PP, Francis BA, Junk AK, Smith SD, Singh K, et al. Pediatric glaucoma surgery: a report by the American academy of ophthalmology. Ophthalmology. 2014;121:2107–15. 25066765 10.1016/j.ophtha.2014.05.010

[3] Anderson DR. Trabeculotomy compared to goniotomy for glaucoma in children. Ophthalmology. 1983;90:805–6. 6622020 10.1016/s0161-6420(83)34484-5

[4] Al-Hazmi A, Awad A, Zwaan J, Al-Mesfer SA, Al-Jadaan I, Al-Mohammed A. Correlation between surgical success rate and severity of congenital glaucoma. Br J Ophthalmol. 2005;89:449–53. 15774922 10.1136/bjo.2004.047761 PMC1772605

[5] Skuta GL, Parrish RK 2nd. Wound healing in glaucoma filtering surgery. Surv Ophthalmol. 1987;32:149–70. 3328315 10.1016/0039-6257(87)90091-9

[6] Sidoti PA, Belmonte SJ, Liebmann JM, Ritch R. Trabeculectomy with mitomycin-C in the treatment of pediatric glaucomas. Ophthalmology. 2000;107:422–9. 10711876 10.1016/s0161-6420(99)00130-x

[7] Wagle NS, Freedman SF, Buckley EG, Davis JS, Biglan AW. Long-term outcome of cyclocryotherapy for refractory pediatric glaucoma. Ophthalmology. 1998;105:1921–7. 9787365 10.1016/S0161-6420(98)91042-9

[8] Budenz DL, Gedde SJ, Brandt JD, Kira D, Feuer W, Larson E. Baerveldt glaucoma implant in the management of refractory childhood glaucomas. Ophthalmology. 2004;111:2204–10. 15582075 10.1016/j.ophtha.2004.05.017

[9] Beck AD, Freedman S, Kammer J, Jin J. Aqueous shunt devices compared with trabeculectomy with Mitomycin-C for children in the first two years of life. Am J Ophthalmol. 2003;136:994–1000. 14644208 10.1016/s0002-9394(03)00714-1

[10] Elhusseiny AM, VanderVeen DK. Outcomes of glaucoma drainage devices in childhood glaucoma. Semin Ophthalmol. 2020;35:194–204. 32567448 10.1080/08820538.2020.1781906

[11] Beck AD, Chang TCP, Freedman SF. “Definition, classification, differential diagnosis.” Childhood Glaucoma: Consensus Series 9. Amsterdam: Kugler; 2013. https://wga.one/consensus/consensus-9/. Accessed 28 Mar 2026

[12] Matsushita K, Kawashima R, Kawasaki R, Nishida K. Prognostic factors for successful Baerveldt glaucoma implant surgery for refractory glaucoma after multiple surgeries. Jpn J Ophthalmol. 2021;65:820–6. 34374907 10.1007/s10384-021-00864-2

[13] Rolim de Moura C, Fraser-Bell S, Stout A, Labree L, Nilfors M, Varma R. Experience with the Baerveldt glaucoma implant in the management of pediatric glaucoma. Am J Ophthalmol. 2005;139:847–54. 15860290 10.1016/j.ajo.2004.12.028

[14] van Overdam KA, de Faber JT, Lemij HG, de Waard PW. Baerveldt glaucoma implant in paediatric patients. Br J Ophthalmol. 2006;90:328–32. 16488956 10.1136/bjo.2005.078832 PMC1856948

[15] Tai AX, Song JC. Surgical outcomes of Baerveldt implants in pediatric glaucoma patients. J AAPOS. 2014;18:550–3. 25456028 10.1016/j.jaapos.2014.08.003

[16] Banitt MR, Sidoti PA, Gentile RC, et al. Pars plana Baerveldt implantation for refractory childhood glaucomas. J Glaucoma. 2009;18:412–7. 19525734 10.1097/IJG.0b013e31818624bd

[17] Vinod K, Panarelli JF, Gentile RC, Tello C, Liebmann JM, Rodriguez N, et al. Long-term outcomes and complications of pars plana Baerveldt implantation in children. J Glaucoma. 2017;26:266–71. 28002192 10.1097/IJG.0000000000000611

[18] Trigler L, Proia AD, Freedman SF. Fibrovascular ingrowth as a cause of Ahmed glaucoma valve failure in children. Am J Ophthalmol. 2006;141:388–9. 16458704 10.1016/j.ajo.2005.08.033

[19] Hill RA, Pirouzian A, Liaw L. Pathophysiology of and prophylaxis against late Ahmed glaucoma valve occlusion. Am J Ophthalmol. 2000;129:608–12. 10844051 10.1016/s0002-9394(99)00465-1

[20] Gedde SJ, Herndon LW, Brandt JD, Budenz DL, Feuer WJ, Schiffman JC, et al. Postoperative complications in the Tube Versus Trabeculectomy (TVT) study during five years of follow-up. Am J Ophthalmol. 2012;153:804–14. 22244522 10.1016/j.ajo.2011.10.024 PMC3653167

[21] Miyako F, Kiuchi Y, Onoe H, Okada N, Okumichi H, Hirooka K. A case of intraocular proliferative changes caused by a glaucoma tube device. Cureus. 2022;14:e26445. 35915674 10.7759/cureus.26445 PMC9338429

[22] Tsai JH, Freeman JM, Chan CC, Schwartz GS, Derby EA, Petersen MR, et al. A progressive anterior fibrosis syndrome in patients with postsurgical congenital aniridia. Am J Ophthalmol. 2005;140:1075–9. 16376654 10.1016/j.ajo.2005.07.035

