# Supplementary material for: Fibrotic response after Baerveldt glaucoma drainage device surgery: an initial sight-threatening sign in eyes with refractory childhood glaucoma
Source: Jpn J Ophthalmol. 2026 May 2;70(4):923–30. doi: 10.1007/s10384-026-01357-w (PMC13452745; doi:10.1007/s10384-026-01357-w)
Supplement: Supplementary file 1 [file 10384_2026_1357_MOESM1_ESM.pdf]

Fibrotic response after Baerveldt glaucoma drainage device surgery: an initial sight-threatening sign in eyes with refractory childhood glaucoma

Rumi Kawashima

Online Resource 1.

The time course of the IOP and IOP-lowering medication scores

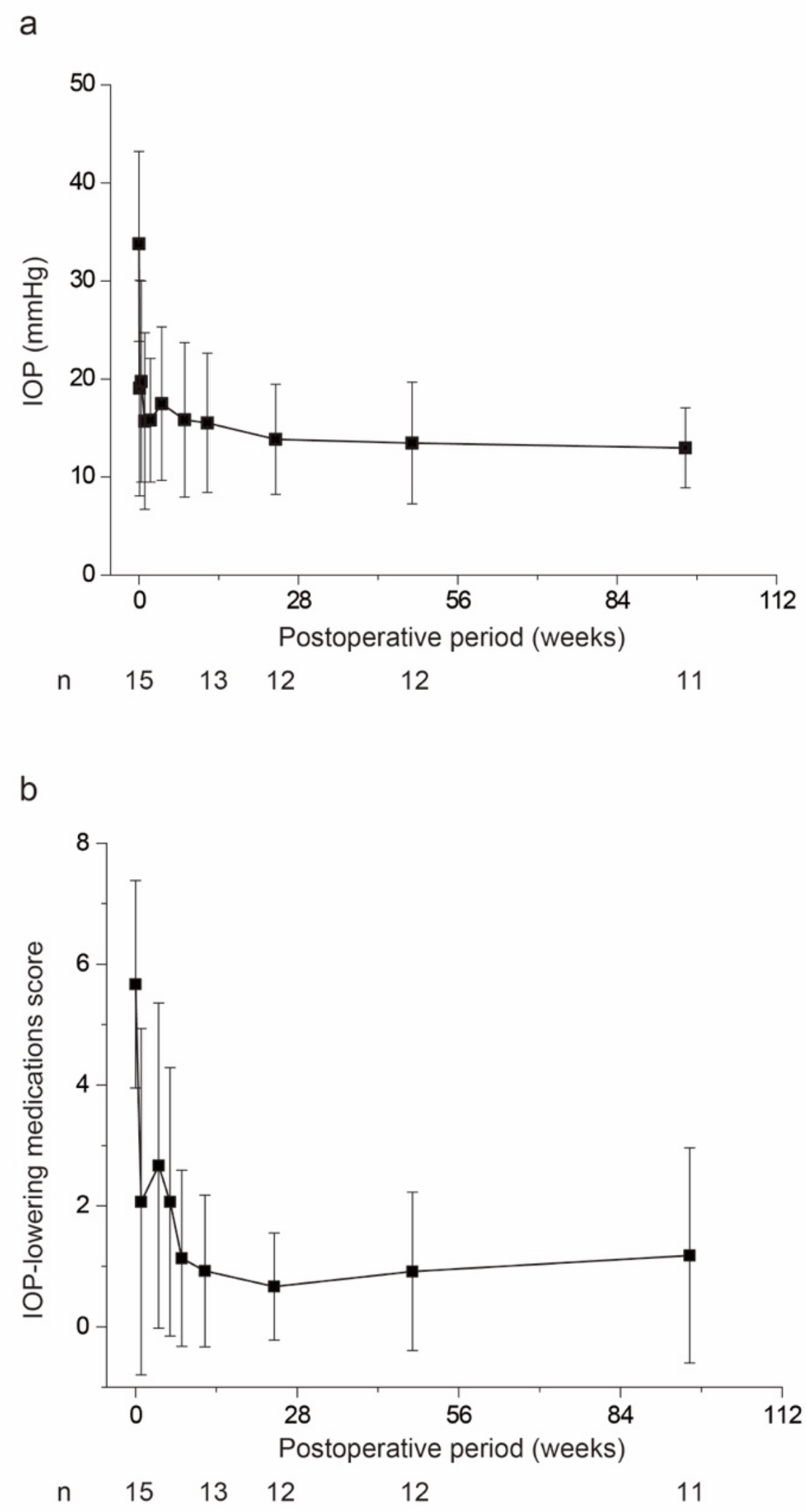

(a) The IOP time course after BGI surgery. The IOP decreased significantly ($P < 0.0001$, repeated analysis of variance [ANOVA] test, and Dunnett's test) during the follow-up period.

(b) The IOP-lowering medication scores after BGI surgery. The IOP-lowering medication

score was defined as one point for each IOP-lowering eyedrop and two points for the oral medication (acetazolamide) daily. The glaucoma eyedrop score decreased significantly ($P < 0.0001$, repeated ANOVA test, and Dunnett's test) during follow-up. The error bars represent the standard deviation (SD). The mean IOP decreased preoperatively to 19.3 ± 8.2 mmHg at 2 years ($P = 0.001$ by repeated ANOVA). The mean IOP-lowering medication score after BGI surgery decreased preoperatively to 1.2 ± 1.8 ($P< 0.0001$ by repeated ANOVA). The mean IOP and IOP-lowering medication score were significantly lower than the preoperative IOP and IOP score ($P < 0.01$ by Dunnett's test)

Online Resource 2.

Survival curves after BGI surgery

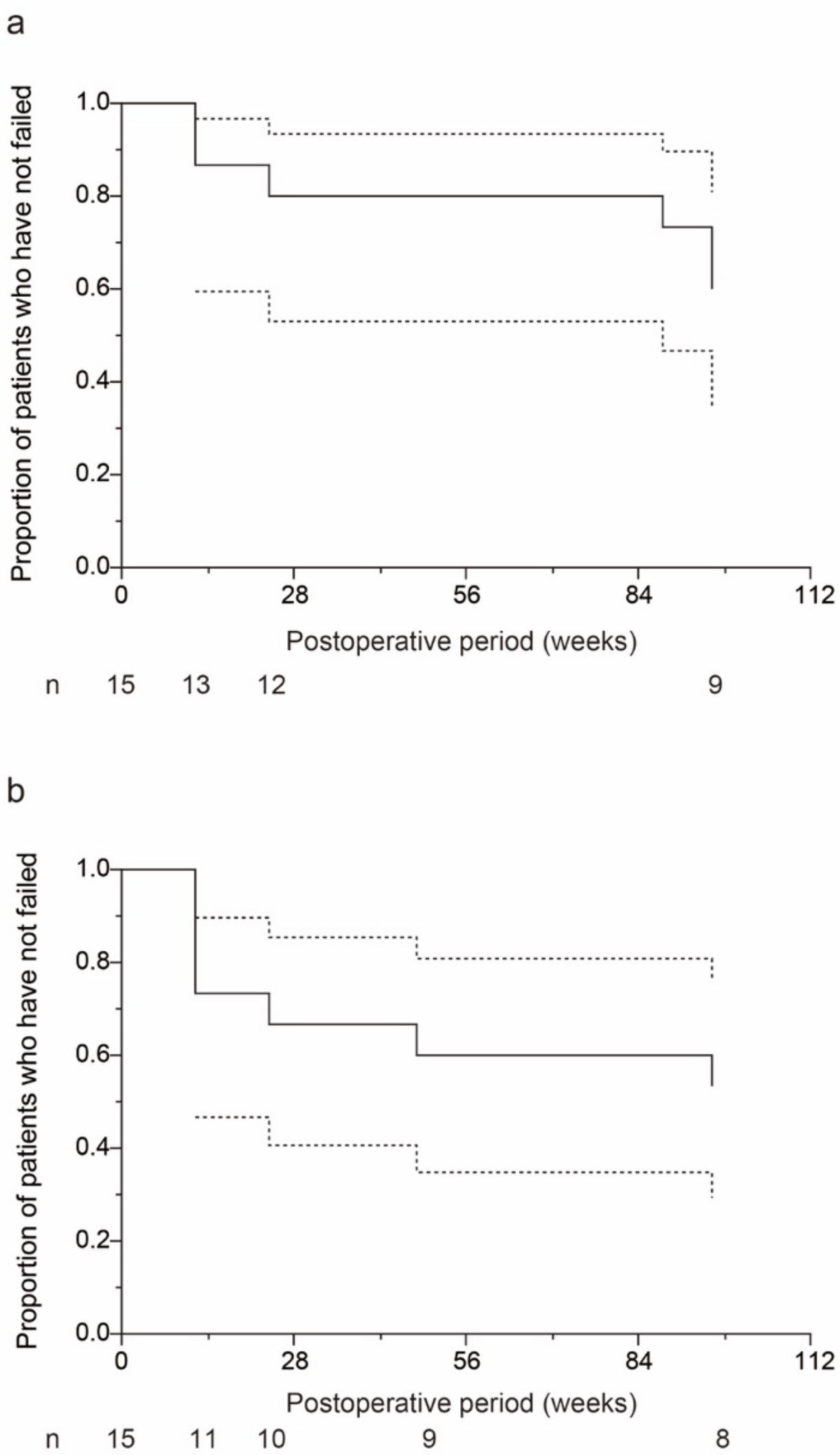

(a) According to failure definition 1. (b) According to failure definition 2. We defined failure as the need for additional IOP-lowering surgery, loss of light perception or postoperative complications comparable to no light perception during the follow-up period, and a postoperative IOP criteria. Regarding the postoperative IOP, we defined failure as an IOP

exceeding the following criteria at two consecutive visits after BGI surgery with or without IOP-lowering medications: definition 1, IOP ≧22 mmHg, and definition 2, IOP ≧17 mmHg. We analyzed the success rate using the Kaplan-Meier method and log-rank test. The dotted lines show the 95% confidence intervals. Based on failure definition 1, the success rates were 80.0% at 12 months and 60.0% at 24 months after BGI surgery, and based on failure definition 2, 60.0% at 12 months and 53.3% at 24 months after BGI surgery
